# High Cardiovascular Disease Risk Among Men and Women Experiencing Poverty and Homelessness in Medically Underserved Areas of West Texas

**DOI:** 10.3390/healthcare13182303

**Published:** 2025-09-15

**Authors:** Eli Heath, Abdulkader Almosa, Rebecca Joseph, Duke Appiah

**Affiliations:** 1 Department of Chemical Engineering, Texas Tech University, Lubbock, TX 79409, USA; eliheath@ttu.edu; 2 College of Arts and Sciences, Texas Tech University, Lubbock, TX 79409, USA; aalmosa@ttu.edu; 3 School of Medicine, Texas Tech University Health Sciences Center, Lubbock, TX 79430, USA; becky.joseph@ttuhsc.edu; 4 Julia Jones Matthews School of Population and Public Health, Texas Tech University Health Sciences Center, Lubbock, TX 79430, USA

**Keywords:** cardiovascular disease, women, men, poverty, homeless, underserved, Texas

## Abstract

Background: While women are among the fastest-growing subgroups of the homeless population and have a higher prevalence of poverty than men, most studies of cardiovascular disease (CVD) risk in this vulnerable population are conducted primarily among men. We evaluated differences in CVD risk between men and women experiencing poverty and homelessness in West Texas, a medically underserved region. Methods: Data were collected from 152 adults (50% women) aged 30–74 years, who were seen at free health clinics. Prevalence ratios (PR) and 95% confidence intervals (CI) were calculated. The Framingham risk algorithm was used to estimate the risk for incident CVD in the next 10 years. Results: The mean age of participants was 55.3 years. The prevalence of CVD risk factors was high among participants and tended to be similar between men and women: diabetes (34%), current smokers (47%), obesity (50%), and hypertension (83%). After controlling for demographic factors, behavior/lifestyle factors, and health conditions, the high (>20%) 10-year risk of CVD was two-fold higher among men (PR: 2.41, 95%CI: 1.75–3.32). While the association was consistent among men regardless of the number of comorbid conditions, among women, those with three or more comorbid conditions had an elevated 10-year risk for CVD compared to those with no comorbid conditions (PR: 11.68, 95%CI: 1.88–72.60, p interaction = 0.001). Conclusions: This study found that CVD risk was elevated among poor and homeless adults, with men having a higher risk than women. Comorbid conditions had a greater impact on women than men who were at high risk for CVD.

## 1. Introduction

Homelessness and poverty, which affect all ages, races, ethnicities, sexes, and genders, are growing problems in several rural and urban locations in the United States [1]. Several reports show that homeless individuals and people with low socioeconomic status are at high risk for chronic diseases, including cardiovascular disease (CVD) [1,2,3,4,5]. Homelessness and poverty often affect health through a dynamic interaction of intrinsic factors (adverse childhood experiences, lack of education, unemployment, family breakdown, disability, mental illness or substance abuse, higher rates of comorbidities and risk factors) and extrinsic factors (social and environmental elements including lack of access to affordable healthcare, food insecurity, limited public benefits, and discrimination) [1,2,6,7].

Homelessness and poverty may confer elevated risks for CVD as a constellation of factors related to biologic (high blood pressure, dyslipidemia, and metabolic syndrome), behavioral (smoking, excessive alcohol intake, physical inactivity and poor dietary practices), and psychosocial (stress and depression) processes, which often interface with structural factors like limited access to healthcare and are reported to be high in these vulnerable populations [8]. Furthermore, population-level CVD screening efforts often exclude people experiencing homelessness and individuals with low income [9].

Women tend to be more vulnerable to the detrimental effects of poverty and homelessness compared to men; however, the cardiovascular implications of poverty and homelessness are less studied in this population. Despite the well-documented sex and gender differences in the prevalence of homelessness and poverty in the United States [10,11], the majority of the available evidence regarding CVD risk among homeless individuals, who are typically of low socioeconomic status, is derived from studies with a largely male cohort [1,2,3,4,5,12]. In instances where differences in CVD risk between men and women have been considered, the evidence has been inconclusive [4,5,7,13,14].

Underserved populations are at high risk for poverty and homelessness, and are predisposed to adverse cardiovascular outcomes [15]. Underserved populations include medically underserved areas, which are areas designated by the United States federal government as having a shortage of primary healthcare services [16]. Such areas are also known to have high rates of poverty and homelessness [16]. Although it is likely that the cardiovascular effects of homelessness and poverty will be substantial for individuals living in such resource-limited settings, investigations of the relationship between homelessness, poverty, and CVD in medically underserved areas are scarce.

Homelessness is reported to be at an all-time high in the United States [17], and women are among the fastest-growing subgroups of the homeless population [18]. Furthermore, women are also reported to have a higher prevalence of poverty than men [10]. Therefore, the aim of the study was to evaluate differences in the future risk for CVD between men and women who were homeless or had low socioeconomic status in the city of Lubbock. This city is in the large West Texas region, which is predominantly a medically underserved region of Texas.

## 2. Materials and Methods

### 2.1. Study Population

The Ark of Hope Foundation is a non-profit organization that provides preventive medical services to underserved communities across the United States. The Raider Medical Screening Society, which is the Lubbock, Texas, branch of this foundation and is affiliated with Texas Tech University, provides free health clinics across the city. Lubbock city is the county seat of Lubbock County, which is classified as a medically underserved population [16]. Data for the current cross-sectional study is from individuals who were seen at these free health clinics located at one homeless shelter and two non-profit social service centers found in zip codes characterized as low income. Thus, a significant proportion of individuals in these zip codes have a median household income below 150% of the federal poverty level [19]. Of the 195 individuals aged 30 to 74 years (the age limits for the Framingham risk score equation) seen at these free health clinics between 1 January 2022 and 30 April 2024, 21 individuals with a self-reported history of CVD and 22 individuals with missing values for covariates used in calculating the 10-year CVD risk were excluded. This resulted in an analytic sample of 152 (76 men and 76 women). The current study was approved by the Institutional Review Board of the Texas Tech University Health Sciences Center (IRB-FY2024-273 on 9 September 2024).

### 2.2. Measures

Data from the first visit was used for this study. Demographic and behavior/lifestyle factors, as well as history of prevalent health conditions, were all self-reported at the time of the clinic visit. Health conditions reported included cancer, chronic renal disease, hypertension, type-2 diabetes, hyperlipidemia, neurological disorder, and mental health illness. Standardized clinical procedures were used to obtain random measures of blood glucose and lipids from finger-stick blood samples. Systolic blood pressure was measured twice using a random-zero sphygmomanometer, with the average of the two used for analysis. Hypertension was defined as a mean systolic blood pressure ≥130 mmHg, a diastolic blood pressure ≥80 mmHg, or a self-reported history of a physician diagnosis of hypertension. Since adherence to treatment for hypertension has been reported to be suboptimal among vulnerable populations like the homeless and people with low-income status [20], controlled hypertension was used as a proxy for current use of hypertension medication, and it was defined as having blood pressure <130/80 mmHg among individuals who reported a history of hypertension. Diabetes was defined as a self-reported physician diagnosis, with random glucose measures ≥200 mg/dL or hemoglobin A1C levels ≥6.5%. Obesity was defined as a body mass index (BMI) ≥30 kg/m^2^.

The risk of a CVD event over the next 10 years was estimated using the non-laboratory Framingham CVD risk equation. Owing to prior reports indicating infeasibility in obtaining fasting blood samples in homeless populations [12,21], coupled with the possibility that some lipid measures among participants in the current study may not have been obtained in a fasting state, the non-laboratory Framingham CVD risk equation was used. Age, sex, systolic blood pressure, treatment for hypertension, current smoking status, diabetes, and BMI are the inputs for the risk equation [22]. The 10-year cumulative risk ranged from 0 to 100%, with scores from 0% to 9% considered low risk, 10% to 20% considered intermediate risk, and >20% considered high risk for incident CVD events over the next decade [22].

### 2.3. Sample Size Estimation

To estimate the minimum sample size for the study, the formula for detecting differences between two proportions, with continuity correction, was employed using the piface software [23]. In a prior study conducted in the United States, the prevalence of increased 10-year CVD risk was reported to be 22% in individuals in low socioeconomic communities and 34% among individuals living in homeless shelters [24]. Furthermore, the prevalence of elevated CVD risk is reported to be 2 or more times higher in men compared to women [25]. Therefore, assuming a prevalence of high CVD risk of 28% in women and 56% in men, and considering an equal number of men and women, an alpha of 0.05 and power of 80%, 61 men and 62 women were needed to detect the aforementioned meaningful differences in CVD risk. Anticipating the possibility that, at most, a quarter of the sample to be recruited will have missing values for components used in estimating CVD risk, a minimum sample of 152 individuals will be needed in order to detect significant differences between men and women in such a situation.

### 2.4. Statistical Analysis

Characteristics of the population were described using means (standard deviation), median (interquartile range), and proportions. Differences in these characteristics between men and women were determined using t tests for quantitative variables with a normal distribution, the Wilcoxon rank sum test for quantitative variables with a non-normal distribution, and chi-square tests for qualitative variables. Owing to the high proportion of participants with a high 10-year risk for CVD (the outcome for the current study), we estimated prevalence ratios (PR) using generalized linear models assuming a Poisson distribution and a log link function with the robust sandwich variance estimator implemented. Models were first adjusted for age and race/ethnicity, and later further adjusted for current use of alcohol and the number of comorbidities reported at the first clinic visit. Multiplicative interactions between gender, age, race and ethnicity, and number of comorbidities on the high 10-year risk of CVD were also tested. Statistical significance was determined with *p* values less than 0.05.

## 3. Results

Overall, the mean age of the sample was 55.3 years (standard deviation: 11.9), with 50% of them being women. The racial and ethnic distribution of the sample was as follows: Non-Hispanic White (29%), Non-Hispanic Black (12%), Hispanic (57%), and 2% of “Other” race and ethnicity. Almost half of the participants reported being current smokers, 15% reported current use of alcohol, and two-thirds of the sample had at least one comorbidity at the first clinic visit. The prevalence of diabetes (34%), smoking (50%), obesity (50%), and hypertension (83%) was very high in this sample. Characteristics of the sample for men and women are provided in Table 1.

**Figure 1:**
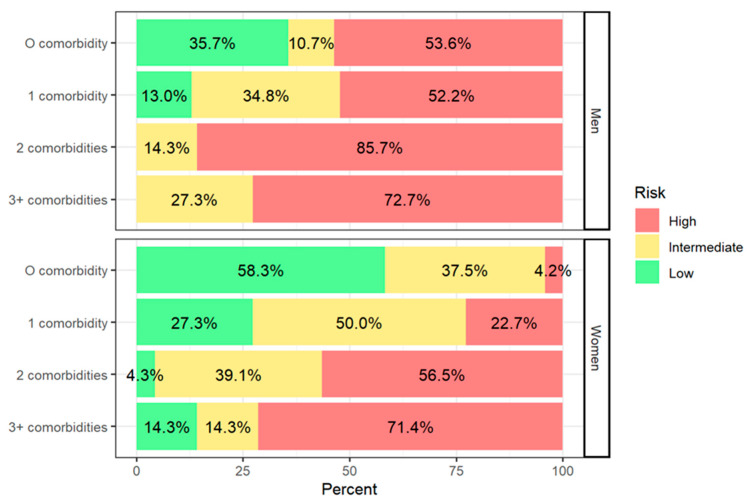
The 10-year risk for cardiovascular disease among men and women experiencing poverty and homelessness by comorbidity status.

Of note, a greater proportion of men consumed alcohol compared to women (22.4% vs. 9.2%). While there were differences in the distribution of other sample characteristics between men and women, such as age, race and ethnicity, smoking status, body mass index, hypertension, diabetes, and number of comorbidities, they did not approach statistical significance. The median (interquartile range) 10-year risk of CVD was 18.9% (10.6%, 35.2%), which was twice as high in men than in women. Furthermore, the proportion of high 10-year risk of CVD was greater among men compared to women (61.8% vs. 31.6%) and among individuals with comorbidities (Figure 1).

In models adjusted for age and race/ethnicity, the prevalence of high 10-year risk of CVD was almost 125% higher among men than women (Table 2). Additional adjustment for alcohol consumption and number of comorbid conditions at first visit increased the estimate (PR: 2.41, 95% CI: 1.75–3.32). There was a significant interaction between gender and number of comorbid conditions, indicating that the effect of comorbid conditions on high 10-year risk for CVD was different across genders (*p* = 0.001).

**Figure 2:**
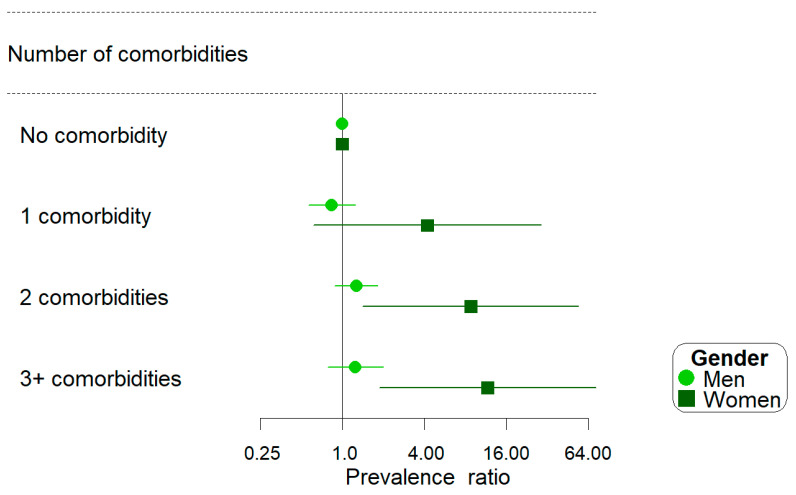
Prevalence ratios for high 10-year risk for cardiovascular disease among men and women experiencing poverty and homelessness by comorbidity status.

While the high risk for CVD over the next 10 years was similar across levels of comorbid conditions in men (*p* = 0.137), a different pattern was observed among women (*p* = 0.007). Compared to women with no comorbid condition, those with three or more comorbid conditions had an elevated risk for high CVD risk over the next 10 years (PR: 11.68, 95%CI: 1.88–72.60) (Figure 2). Among individuals with three or more comorbidities, there was no difference in the high 10-year risk for CVD between men and women (PR = 0.73, 95%CI: 0.44–1.22, *p* = 0.230), indicating a greater impact of comorbidities on CVD risk in women compared to men. No statistically significant interactions were found for age and race/ethnicity.

## 4. Discussion

In this study of adults with low socioeconomic status or experiencing homelessness from a medically underserved population, men had a two-fold elevated risk of high CVD risk over the next 10 years. This association varies by the number of co-morbidities. Among women, the risk of a high 10-year CVD risk increased with a greater number of comorbid conditions. In contrast, among men, the 10-year risk for CVD remained relatively stable across increasing comorbidity burden.

The majority of studies, especially among homeless individuals, have predominantly included men with limited representation of women. As such, there have been few studies comparing CVD risk between women and men who are homeless and of low socioeconomic status. Even in studies with good representation of women, gender or sex differences have often not been considered [5,13]. Among the few studies that considered differences in CVD risk between men and women, the evidence has been inconclusive [4,5,7,13,14,26]. In our study, which attempts to bridge current gaps by exploring gender-based differences in future risks for CVD among a uniquely vulnerable population from a low-income community with a high proportion of participants reporting homelessness, women were observed to have a higher likelihood of hypertension than men [14]. However, these results were not adjusted for potential confounders. Results from a recent meta-analysis of 12 studies found similar elevated risk for CVD morbidity and mortality in homeless males and females compared to housed populations [7]. Another meta-analysis of 116 cohorts primarily from high-income countries also reported no sex difference in the risk for coronary heart disease and stroke for individuals with low income [27]. However, a higher excess risk for CVD was observed among females compared to males when a composite measure of socioeconomic status comprising education, occupation, income, or area of residence was used [27]. In the current study, men had a greater risk of high CVD risk over the next 10 years. Interestingly, baseline data did not indicate many significant differences between men and women, which may potentially be due to the relatively small sample size of the study population.

About 77% of adults experiencing homelessness are reported to have one or more chronic health conditions [28]. While the early and more severe onset of chronic health conditions in this population is reported to contribute to elevated risk of premature mortality [28], the impact of the number of comorbidities on the risk of CVD is not well known. In the current study, in which two-thirds of participants had at least one comorbidity, the findings suggest that among poor and homeless adults, women with higher levels of comorbidities have a higher risk of CVD than women with no comorbidities, and this risk was similar to men with higher levels of comorbidities. With women experiencing homelessness and poverty reported to have greater comorbidities than men, including cardiometabolic and mental health conditions [29,30,31], the finding of this current study is reflective of the fact that in the general population, these comorbidities have a higher impact on CVD in women compared to men [32,33]. Given that healthcare disparities among poor and homeless adults are particularly distressing for women [34], coupled with women experiencing homelessness in the United States having an average of 3.5 health conditions [35], there is a critical need for more targeted interventions for CVD prevention among this vulnerable subgroup of the population to enhance their quality of life.

Despite homeless and poor adults being a diverse group based on reasons and causes of their current state of being, they share a common vulnerability to morbidity than the general population. Regardless of gender, we observed in the current study a high burden of CVD risk factors that demand critical attention, especially as these individuals are from a medically underserved population. For instance, the prevalence of diabetes, obesity, and hypertension was extremely high at 34%, 50%, and 83%, respectively. Furthermore, almost half of the study population were predicted to have a cardiovascular event in the next 10 years if interventions are not implemented. Potential factors contributing to this high CVD risk factor burden include lack of access to affordable preventive care, late presentation or fragmented healthcare care, and competing psychosocial priorities [4].

With regard to mechanisms, several have been proposed to explain the difference in CVD risk between men and women. Men are reported to have a greater burden of adverse CVD risk profiles than women, with behavior and lifestyle factors such as smoking, alcohol intake, and diet implicated in this pattern [36]. With adverse CVD risk profiles often leading to a more pro-inflammatory state, several histological and imaging studies have shown a greater overall burden of inflammation, inflammation-induced endothelial dysfunction, and atherosclerotic plague in men than women [37,38]. Genetic and hormonal factors play important roles in inflammation [39,40]. As such, women tend to achieve resolution of inflammation quicker than men as they often have greater production of anti-inflammatory cytokines [41]. Emerging evidence also shows variation in the expression of the sex chromosomes that influence several processes related to CVD risk factors and overt adverse cardiac outcomes [42]. Although debated, some studies provide considerable evidence of the direct favorable effects of endogenous female sex hormones like estrogen and the expression of estrogen receptors on cardiac physiology and function [43]. Besides these biological factors, structural factors such as healthcare access also influence differences in high CVD risk between men and women. For instance, on average, a greater proportion of women are reported to have healthcare coverage, a primary care physician, and routine checkups compared to men, although they also often encounter delays in healthcare access or unmet healthcare needs, especially among young women [44]. While this observation may not necessarily translate to the homeless population, men who are homeless are reported to be less likely to seek healthcare than homeless women, who often benefit from healthcare service utilization while still experiencing significant health barriers compounded by social and economic factors [45,46,47].

Taken together, the findings of the current study stress the importance of ensuring accessible healthcare for poor and homeless individuals, as well as the implementation of long-term supportive housing and income assistance interventions, which have been shown to yield considerable successes in improving the livelihood of vulnerable populations [48]. Additionally, there is urgent need for increased public health efforts through public–private sector partnerships and community–academic partnerships to support free clinics and hospitals to improve the health outcomes of underserved populations, especially the most vulnerable subgroups such as the poor and homeless. At the population level, implementing policies that address social determinants of health and reduce extrinsic causes of poverty and homelessness, such as expanding affordable housing, community support services, and social safety nets, will go a long way to reduce the high burden of CVD morbidity and mortality among poor and homeless individuals [49].

Beyond the strengths of the current study, which includes a sample of homeless and poor adults with a good balance between men and women, as well as characterized CVD risk profiles, the following limitations deserve consideration. First, the sample was relatively small, which may have prevented the detection of small differences in CVD risk factors between men and women. Along the same lines, the relatively small sample size resulted in imprecise estimates, especially for the interaction effects, as evidenced by wider confidence intervals for women. Second, due to the nature of the study population, fasting measures of lipids could not be obtained for the entire sample as the free clinics operated on a walk-in basis. Third, information on the frequency and duration of homelessness was not available. Other studies have observed a positive association between the duration of homelessness and CVD risk factors [21], which suggests that this information may be important for risk stratification in this population and for informing robust intervention strategies. Also, other information like education, income, marital status, and annual household income was not obtained in the current study due to the vulnerable nature of the population and to assure them of their privacy [50]. Fourth, the potential for residual confounding influencing these results cannot be entirely ruled out due to the limited covariates adjusted in the multivariable models. Fifth, some comorbidities and other factors like homelessness were self-reported, potentially resulting in non-differential misclassification that often, but not always, biases estimates to the null. With substantial differences observed in CVD risk, coupled with the significant effect of comorbidities on this association, the influence of this misclassification on the current results may be minimal. Finally, vulnerable populations like homeless individuals face several barriers to healthcare, such as cost, stigma, and lack of transportation [51,52]. While free clinics often ameliorate to a large extent barriers related to cost and stigma, they do not entirely address barriers related to the lack of transportation. In the current study, we strived to ensure proximity of the free clinic to the targeted population by situating the clinics in homeless shelters and non-profit social service centers. Despite these efforts, we were not able to target all low-income and homeless individuals in the city. Thus, if the most critically ill homeless individuals did not patronize services from these free clinics, it could lead to underreporting of the risk of CVD, which was observed to be substantially high in the current study. While the specific triggers of homelessness in West Texas may not be the same as in other regions of Texas and other countries, homeless populations across the globe do encounter significant hardships such as poverty, inadequate access to care, lack of affordable housing, and high burden of comorbid conditions [53,54]. Therefore, the findings of the current study, to a large extent, may be generalizable to low-income and homeless populations located in other parts of the country and beyond.

## 5. Conclusions

This study of poor and homeless adults from a medically underserved population showed that, overall, men have a two-fold higher 10-year risk of CVD than women. This association was modified by preexisting comorbid conditions: among women, those with three or more comorbid conditions had an elevated risk of CVD compared to those without comorbid conditions. This high 10-year risk of CVD in women with three or more comorbidities is similar to the estimates for men with three or more comorbid conditions. To adequately quantify differences in the high risk of CVD by sex and the influence of comorbidities on this association in this vulnerable population, confirmation of these findings in larger prospective cohorts of poor and homeless individuals in resource-limited settings is warranted.

## Figures and Tables

**Table 1:** Characteristics of the sample of men and women experiencing poverty and homelessness.

Characteristics ^a^	Women (n = 76)	Men (n = 76)	*p* Value
Age, years	56.3 (12.4)	54.4 (11.4)	0.315
Age group			0.411
30–44 years	16 (21.1%)	16 (21.1%)	
45–64 years	35 (46.1%)	42 (55.3%)	
65–74 years	25 (32.9%)	18 (23.7%)	
Race and ethnicity			0.130
Non-Hispanic White	20 (26.3%)	24 (31.6%)	
Non-Hispanic Black	7 (9.2%)	11 (14.5%)	
Hispanic	49 (64.5%)	38 (50.0%)	
Other	0 (0.0%)	3 (3.9%)	
Current smoker	30 (39.5%)	41 (53.9%)	0.074
Current alcohol user	7 (9.2%)	17 (22.4%)	0.026
Body mass index, kg/m^2^	30.1 (6.4)	30.5 (7.1)	0.752
Obesity	41 (53.9%)	35 (46.1%)	0.330
Systolic blood pressure, mmHg	131.4 (14.0)	136.3 (20.2)	0.084
Hypertension	60 (78.9%)	66 (86.8%)	0.196
Diabetes	29 (38.2%)	22 (28.9%)	0.229
Random total cholesterol, mg/dL ^b^	162.0 (138.0, 184.0)	151.0 (113.0, 181.0)	0.142
Random triglycerides, mg/dL ^b^	112.5 (85.5, 143.5)	110.0 (77.0, 157.0)	0.750
Number of comorbidities			0.333
0	24 (31.6%)	28 (36.8%)	
1	22 (28.9%)	23 (30.3%)	
2	23 (30.3%)	14 (18.4%)	
≥3	7 (9.2%)	11 (14.5%)	
Mean 10-year risk of CVD, %	13.9 (7.2, 22.9)	26.7 (14.5, 45.2)	<0.001

^a^ Values are frequency (%), mean (standard deviation), or median (Interquartile range). ^b^ Differences tested using Wilcoxon rank sum test.

**Table 2:** Prevalence ratios for high 10-year risk of CVD (>20%) among adults experiencing poverty and homelessness, comparing men to women (referent).

Models	PR (95% CI)	*p* Value
Unadjusted	1.96 (1.35–2.85)	<0.001
Model 1	2.25 (1.64–3.10)	<0.001
Model 2	2.41 (1.75–3.32)	<0.001

CI: confidence interval; PR: prevalence ratio. Model 1: adjusted for age and race/ethnicity. Model 2: model 1 plus current alcohol use and number of comorbidities at first visit.

## Data Availability

Dataset available upon request from the authors.

## Abbreviations

The following abbreviations are used in this manuscript:

BMI	Body mass index
CI	Confidence intervals
CVD	Cardiovascular disease
PR	Prevalence ratio
